# Arthroscopic treatment of chondral defects in the hip: AMIC, MACI, microfragmented adipose tissue transplantation (MATT) and other options

**DOI:** 10.1051/sicotj/2017029

**Published:** 2017-06-07

**Authors:** Eugenio Jannelli, Andrea Fontana

**Affiliations:** 1 Clinica Ortopedica e Traumatologica – IRCCS Policlinico San Matteo, Università degli Studi di Pavia 27100 Pavia Italy; 2 Ortopedia 1, COF Lanzo Hospital 22024 Lanzo d’Intelvi (CO) Italy

**Keywords:** Chondral defects, Hip arthroscopy, Debridement, MACI, AMIC

## Abstract

Chondral lesions are currently considered in the hip as a consequence of trauma, osteonecrosis, dysplasia, labral tears, loose bodies, dislocation, previous slipped capital femoral epiphysis and Femoro-Acetabular-Impingement (FAI). The management of chondral lesions is debated and several techniques are described. The physical examination must be carefully performed, followed by radiographs and magnetic resonance imaging (MRI). Differential diagnosis with other pathologies must be considered. Debridement is indicated in patients younger than 50 years with a chondropathy of 1st or 2nd degree. Microfractures are indicated in patients younger than 50 years with a chondropathy of 3rd or 4th degree less than 2 cm^2^. Matrix-Induced Autologous Chondrocyte Implantation (MACI) and Autologous Matrix-Induced Chondrogenesis (AMIC) procedures are indicated in patients with full-thickness symptomatic 3rd–4th degree chondral defects, extended 2 cm^2^ or more. The AMIC procedure has the advantage of a one-step procedure and much less expense. Microfragmented adipose tissue transplantation (MATT) is indicated for the treatment of delamination and 1st and 2nd degree chondral lesions, regardless of the age of the patient. Chondral defects are effective when the joint space is not compromised. When the Tonnis classification is two or greater, treatment of chondral lesions should be considered ineffective.

## Introduction

Chondral lesions are currently considered in the hip as a consequence of other pathological features such as trauma, osteonecrosis, dysplasia, labral tears, loose bodies, dislocation, previous slipped capital femoral epiphysis and Femoro-Acetabular-Impingement (FAI) [1, 2]. Recently, FAI has been indicated as a cause of progressive degenerative changes in the hip, leading to osteoarthritis [3, 4]. The altered morphology of the femur and/or of the acetabulum leads to an abnormal contact against the joint, thereby causing stress degeneration of the labrum and cartilage.

Labral tears have been indicated as an adjunctive cause of cartilage degeneration. Chondral damages have been described for up to 73% of the patients with labral pathology [5–7].

In the FAI cam type, the bone deformity located at the femoral head-neck junction, when forced into the joint, leads to increased friction on the cartilage and on the labral structures. The labrum is pushed up and the stress forces concentrate at the level of the chondrolabral junction leading to a separation of the cartilage from the subchondral bone. The cartilage is pulled and sheared with a “carpet-like” pattern, usually at the antero-superior acetabular region. It was proposed that there is a continuum of damage, which starts with the chondrolabral lesion, then proceeds with the cartilage delamination and finally labral detachment from the subchondral bone [6].

The second type of FAI, the pincer type, is determined by an altered acetabulum, usually as a slight acetabular retroversion or as an overcoverage of the acetabular wall. In this case, there is a large stress impact on the labrum which usually degenerates, tears and sometimes ossifies. As a consequence of pincer deformity, the chondral acetabular lesion is a typical “counter-part” degeneration of the postero-inferior area, or a chondral lesion on the anterior and superior area of the acetabulum, consequent to shear forces concentrating on the chondrolabral junction.

Chondropathies of the acetabulum and the femoral head are a frequent cause of pain and functional limitation. Moreover, if cartilage defects in the hip are not adequately repaired, then progression of the damage and arthritic changes may occur [3, 4].

## Patient selection and examination

Candidate patients for hip arthroscopy must be carefully selected [7], particularly when chondral damages are suspected. They should have mechanical symptoms or persistent pain despite conservative therapy. The physical examination must be carefully performed with all the known signs and tests, and then followed by radiographs and MRI, and eventually by computed tomography (CT) and ultrasonography [3, 8–10], while the differential diagnosis with other pathologies must be considered [11].

The degree of arthritis can be defined by the Tonnis classification [12] and is usually accompanied with the measurement of the radiographic signs such as the alpha angle [6], the cross-over sign, the coverage of the femoral head and estimation of the joint space [13].

MRI without contrast often fails in the identification of chondral defects of small dimension (less than 1 cm^2^). Therefore, an MRI arthrogram is usually suggested [2]. Nevertheless, MRI arthrograms can be over-interpreted, particularly those concerning labral tears and, in selected cases, an intra-articular injection of 10 cc carbocaine 2% can become useful in determining whether the source of pain is intra- or extraarticular (hip injection test) [13, 14]. In addition, a high percentage of false negatives has been reported for plain radiography, scintigraphy, CT, and MRI [2].

## Surgical treatment

### Debridement

Regardless of the type of treatment of chondral lesions, the first step is an accurate articular debridement.

Four steps have been indicated for a correct debridement [15]: abundant washing of the joint, removal of loose bodies, removal of mechanically irritating cartilage and synovia, limited chondroplasty.

Articular washing could be effective for immediate but not lasting pain relief in an inflamed joint, therefore debridement must be seen more as preparation of the joint for microfractures or chondral grafting than as a finished procedure.

Debridement is usually performed with arthroscopic shavers, sharp curettes, arthroscopic burrs or electrothermal devices. Larger resector blades (5.5 mm) have to be preferred, since the smaller ones can be obstructed by fibrous and cartilage fragments. Unstable cartilage flaps and damaged cartilage are unable to heal autonomously and, on the contrary, are a potential source of further intra-articular damages. Therefore, they must be removed. Accurate exploration with probes must be conducted before their removal in order to save as much cartilage as possible.

### Microfractures

Steadman first developed the microfracture technique for the knee [14] reporting very good results for the treatment of full-thickness chondral defect [16, 17].

The technique is based on the penetration of the subchondral bone plate and the consequent outflow of bone marrow blood, containing mesenchymal stem cells (MSCs). The underlying mechanism is the differentiation of the mesenchymal stem cells (MSCs) in fibrochondrocytes which can produce type I, II and III collagen [18]. Therefore the formation of a fibrocartilaginous tissue is expected, with reduced mechanical properties compared to hyaline cartilage.

The subchondral bone is penetrated for approximately 2–4 mm with an arthroscopic awl (30° and 45° are preferable because of the sphericity of the hip) to create V-shaped holes of 1.5–2 mm diameter. The distance between the holes must be about 3 mm. It is usually suggested to begin the microfracture at the periphery and to proceed towards the centre. In addition, it is important to penetrate the subchondral bone perpendicularly. This can be particularly difficult in the hip, specifically in the supero-anterior areas of the acetabulum. Bone marrow bleeding from the holes must be checked reducing the water pressure or removing the arthroscopic fluid.

The indications for microfracture in the hip are similar to the knee and include focal and contained lesions, typically ≤2 cm^2^ in size.

### Matrix-induced autologous chondrocyte implantation (MACI)

Matrix-Induced Autologous Chondrocyte Implantation (MACI) is a technique described for the treatment of chondral defects of the knee, where it has given good clinical results [19].

This technique requires two surgical steps: the biopsy of cartilage for chondrocyte culture and expansion followed by their direct culture in a membrane used as a scaffold; and a second operation where the seeded membrane is inserted into the joint and applied to cover the chondral defect.

Several materials have been proposed as matrices: protein-based polymers (collagen types I and III, fibrin, gelatin, etc), carbohydrate polymers (hyaluronic acid, polylactic acid, polyglycolic acid, alginate) and artificial polymers [20–23].

Indications for MACI are: full-thickness symptomatic chondral defects, in general of 3rd–4th degree, extended 2 cm^2^ or more in patients 50 years old or younger and with an uncompromised joint space on a standard X-ray (Tonnis less than grade 2).

Absolute contraindications are infections, inflammatory arthritis, tumors, a compromised joint space (Tonnis grade 2–3) and nonadequate patient compliance.

During the first arthroscopic step, the hip is carefully examined and once the decision to perform a MACI is confirmed, a few fragments of full-thickness cartilage (about 5–10 mm) are taken from the area surrounding the pulvinar. Then the samples are sent to laboratories for culturing.

During the second step, after accurate chondrectomy the fluid is completely removed and the membrane is implanted. It is directly inserted into the articular space using an arthroscopic cannula and is then adapted to cover the chondral defect. An accurate chondrectomy with very sharp edges, the concavity of the acetabulum and the pressure of the femoral head against the acetabulum once the traction is released, give the implant sufficient stability.

Implant fixation is an issue and must be controlled. After having positioned the implant on the cartilage defect, it is a good rule to release the traction and to execute a series of 4–6 flex-extension and rotation movements. Then the traction must be re-applied and the position of the membrane verified. In case the membrane does not show acceptable stability, it is possible to utilize fibrin glue to fix it.

### Autologous matrix-induced chondroplasty (AMIC)

This technique combines the potential benefits of a single-step procedure of marrow stimulation with membrane-induced growth. It is based on performing microfractures followed by the implantation of a resorbable collagen membrane (Chondro-Gide^®^, Geistlich Pharma AG) to cover the chondral defect. This membrane has two different surfaces: one is smooth and regular, the other is rough and porous in structure. The rough surface is the one which must be applied against the subchondral microfractured bone. In this case, the bone marrow mesenchymal stem cells (MSCs) are held in situ by the membrane which would lead to a differentiation process towards cartilage-like tissue.

Other advantages of the AMIC procedure are that logistics for transportation of the cells to the laboratory is not required and that the planning of the operation is much easier.

Indications are the same as those described above for the MACI technique.

**Figure 1.**
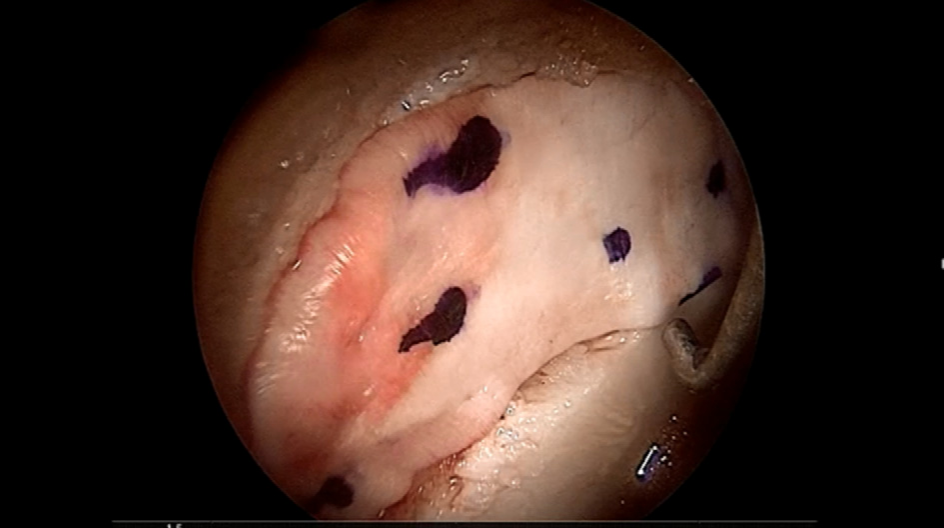
The membrane is applied to cover the acetabular chondral defect, after microfracturing.

During hip arthroscopy, the joint is evaluated and the chondral defect located. After accurate debridement a microfracture treatment is performed. The membrane is then implanted using a technique similar to that described above for the MACI procedure. Care must be taken to properly mark the smooth surface of the membrane with some dots in order to be sure that the rough porous face is correctly applied against the subchondral bone (Figure 1).

In some cases, arthroscopic manoeuvres fail to properly adapt the membrane flat to cover the chondral defect. In those cases, it is suggested to insert into the joint a urinary bladder catheter and to inflate it so that its expansion compresses, becomes flat and stabilizes the membrane against the defect.

Again, the intrinsic stability is usually sufficient, but fibrin glues can also be used to promote fixation.

### Microfragmented adipose tissue transplantation (MATT)

Articular cartilage possesses only a weak capacity for repair; on the other hand, mesenchymal stem cells (MSCs) are specified as appropriate cell candidates for regenerating incurable defects of articular cartilage due to the following characteristics: inherent chondrogenic property, easy availability, cell homing potential and immunomodulatory function [24, 25].

Besides bone marrow, multiple tissues have been reported to contain MSCs. These include adipose tissue [26, 27], trabecular bone [25, 28], synovial membrane [27, 29], skeletal muscles [28, 30]: unlike bone marrow, adipose tissue derived MSCs can be isolated in large quantities with minimal morbidity and discomfort [29, 31].

**Figure 2.**
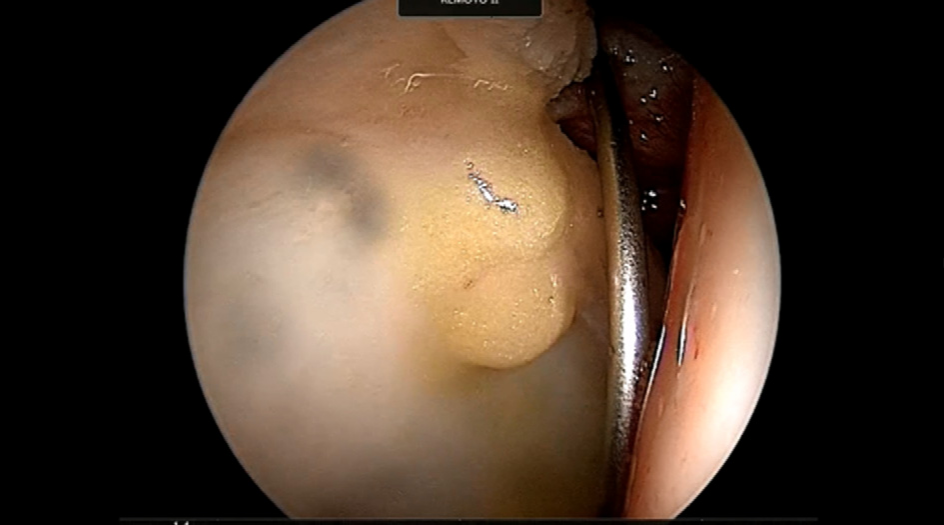
Adipose tissue derived MSCs are injected in between the fibrous chondral layer and the subchondral bone for the treatment of an acetabuar delamination.

Indications for MATT are: delamination or full-thickness symptomatic chondral defects, extended 2 cm^2^ or more with a Tonnis less than grade 2 joint space on a standard X-ray (Figure 2).

As for the AMIC technique, this is a one-step procedure. The autologous adipose tissue is harvested from the subcutaneous area of the lateral proximal thigh (the perithrocanteric area). The cellular component of the tissue is selected and isolated by simply washing with saline solution (Lipogems^®^) and then injected into the joint at the end of the arthroscopic procedure.

## Rehabilitation protocol

Continuous passive motion, from the 1st day post-op, is usually indicated to quickly regain complete range of motion [32, 33].

Full weight bearing is usually contraindicated for about four weeks in patients treated with MACI or AMIC procedure. In these cases, partial weight-bearing exercises are suggested for four weeks.

Biking without resistance is started at day two post-op, as well as open chain exercises to restore gluteal, ischiocrural, adductor, abductor and quadriceps muscles. Swimming and deep water exercises can be started at two weeks after surgery. At four weeks post-op closed chain exercises are introduced. Running and jumping must be avoided for at least three months. Return to agonistic activity is allowed at 6–9 months, depending on the kind of lesion, sports activity and the confidence of the patient.

## Discussion

There is general agreement in the belief that advanced arthritis (narrowing of the articular space inferior than 2 mm) is contraindicated for hip arthroscopy [2, 13]. The issue of the diagnosis is difficult in itself, since accuracy and precision of radiographs and MRI can over- or underestimate the lesions; for this reason, diagnostic arthroscopy itself has still a diagnostic value [2, 3].

Only a few results are reported in the literature on the use of microfractures as a treatment for chondral defects in the hip joint. Nevertheless, today this is a well-established technique, described by several authors as a very promising procedure [34]. Good results have been reported with microfractures for grade IV articular lesions [19], after 2–5 years of follow-up [35, 36].

A high percentage of coverage of the full-thickness (grade IV) chondral acetabular defect has been reported after microfractures even when associated with a kissing femoral lesion [37, 38]. On the contrary, other authors considered cartilage lesions as a limiting factor for significant clinical improvement, particularly those of advanced degree [39].

The treatment of chondral defects with the MACI or AMIC techniques has only been reported for the knee joint, and their application in the hip was exclusively related to personal experiences. For hip chondral lesion management, only two reports were found [30, 31, 40].

Apparently, tri-dimensional matrices allow longer phenotype maintenance of implanted chondrocytes compared to monolayer matrices [41]. This particular aspect of the capacity of the cells to grow in a tri-dimensional way once applied to a membrane is the focal point in new biotechnologies applied to chondral reconstruction. Efforts must be taken in the future to establish the histological type of chondral tissue developed with different operative techniques and scaffolds or cellular culture.

Recently, many new membranes, matrices, biological glue and chondrocyte suspensions have been developed. The goal is a biologically active and stable graft with cartilage-like histological features [23]. Even though the knee still remains the joint with the largest clinical and scientific experiences, the use of such techniques in the hip is of growing interest. Nevertheless, an ex vivo culturing step is necessary for the majority of these techniques, such as BioCart II^®^, Cartilage Autograft Implantation System (CAIS)^®^, Cartilage Regeneration System (CaReS)^®^, Cartipatch^®^, ChondroCelect^®^, DeNovo and MACI^®^, while it is not the case for the autologous matrix-induced chondroplasty (AMIC). Therefore this is a greatly attractive procedure, due to the potential of combining the effects of the marrow stimulation techniques and the benefits of the membrane in guiding differentiation towards cartilaginous tissues [41].

As previously described in this article, chondral defects are frequently associated with other lesions. Actually no studies have been performed to properly define the type, amount and exact location and extension of chondral lesions associated to FAI. Furthermore, labral tears are often associated with chondral defects in the hip and FAI is actually considered as a precursor of primary osteoarthritis [42]. Of course the presence and the treatment of these associated lesions must be considered to obtain good results and long-lasting effects on cartilage treatment [43, 44].

New advanced biotechnologies address the use of autologous MSCs for chondral regeneration. Though this treatment seems to be more effective, surgically simple and reproducible, when compared to the others, still its clinical evidence must be proved.

## Conclusions

The treatment of chondral defects in the hip is still controversial from several standpoints. It must be pointed out that none of the treatments of chondral defects are effective when the joint space is seriously compromised.

All the associated pathologies such as FAI, labral tears, dysplasia etc, must be treated alongside chondral defects.

A decision tree for choosing the appropriate technique for any particular case is reported in Tables 1 and 2.

**Table 1.** Decision tree for patients younger than 50 years.

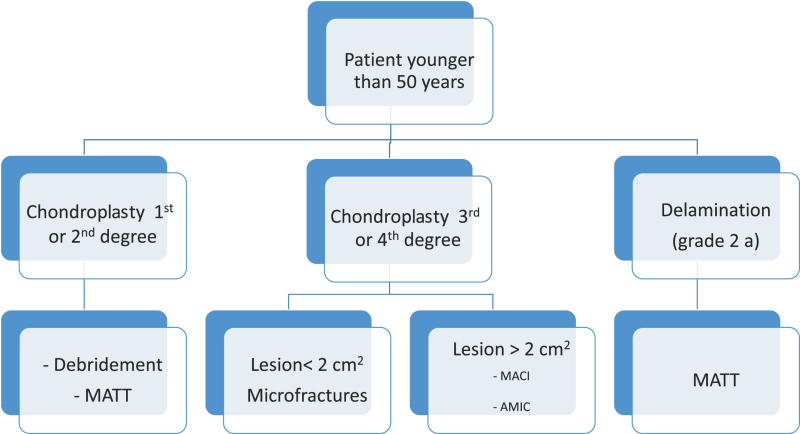

**Table 2.** Decision tree for patients older than 50 years.

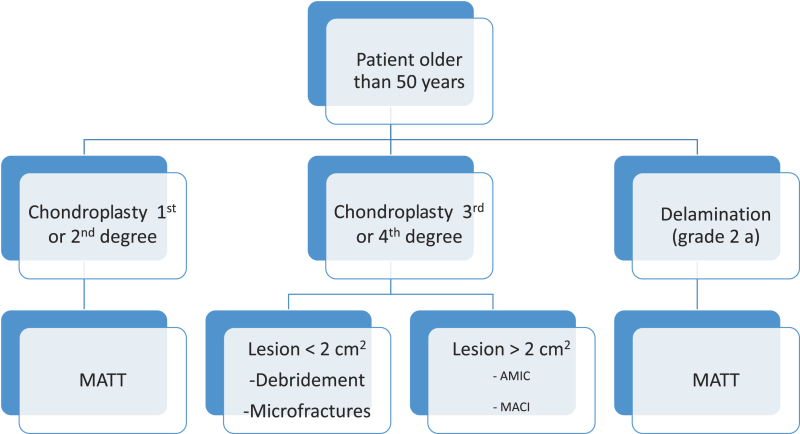

Debridement is indicated in patients younger than 50 years with a chondropathy of 1st or 2nd degree according to the Outerbridge classification, or in patients older than 50 years with a chondropathy of 3rd or 4th degree.

Microfractures are indicated in patients younger than 50 years with a chondropathy of 3rd or 4th degree less than 2 cm^2^ or in patients older than 50 years with a chondropathy of 3rd or 4th degree.

MACI and AMIC procedures are indicated in patients with full-thickness symptomatic 3rd–4th degree chondral defects, extended 2 cm^2^ or more. Best results have been obtained in patients younger than 50 years. The AMIC procedure when compared to the MACI technique has the advantage of a one-step procedure and much less expense.

MATT is indicated for the treatment of early or intermediate chondral lesion such as delamination, regardless of the age of the patient.

## Conflict of interest

The authors declare that they have no conflict of interest.
